# Re-Examining the Agentic Shift: The Sense of Agency Influences the Effectiveness of (Self)Persuasion

**DOI:** 10.1371/journal.pone.0128635

**Published:** 2015-06-08

**Authors:** Tom G. E. Damen, Barbara C. N. Müller, Rick B. van Baaren, Ap Dijksterhuis

**Affiliations:** 1 Utrecht University, Utrecht, the Netherlands; 2 Behavioural Science Institute, Radboud University Nijmegen, Nijmegen, the Netherlands; 3 Ludwig Maximilian University Munich, Department of Psychology, Munich, Germany; Georgetown University Medical Center, UNITED STATES

## Abstract

In the present study we investigated whether differences in the sense of agency influenced the effectiveness of both direct persuasion and self-persuasion techniques. By manipulating both the delay and contingency of the outcomes of actions, participants were led to experience either a low or high sense of agency. Participants were subsequently presented with arguments as to why a clean local environment is important (direct persuasion), or were asked to generate those arguments themselves (self-persuasion). Subsequently, participants’ cleanliness attitudes and willingness to participate in a campus cleanup were measured. The results show that techniques of direct persuasion influenced attitudes and volunteering behavior under conditions of low rather than high agency, whereas techniques of self-persuasion were most effective under conditions of high rather than low agency. The present findings therefore show how recent experiences of agency, a state based experience of control, can influence the effectiveness of both external and internal persuasion techniques.

## Introduction

Our beliefs and feelings about the ability to control or influence our actions, environment, and lives, represent an important aspect of our everyday cognitive experience. In turn, these cognitions can greatly determine our future behavior. Consider for example how important our feelings of control are for the extent to which we form deliberate intentions and set future goals. Would we still plan for the evening, set New Year resolutions, or try out the latest Men’s Health workout for the perfect six-pack without the belief that we are able to make a change? In the present study we investigated how recent experiences of action control, or agency, can shape and determine both our ability to effectuate self-driven change, as well as influence our susceptibility to external forces.

The sense of agency refers to the ability to recognize oneself as the controller of one's own actions and to distinguish these from actions caused or controlled by other sources [1], [2]. When the sense of agency is high we experience a sense of control over our actions. However, when actions are performed without an accompanying sense of agency, it can feel like they are happening to a person instead of being authored by that person [3]. Instead of a long-term belief of personal control, the sense of agency therefore represents a state construct that binds together our thoughts, actions, and action-effects to give rise to the personal sense of having successfully influenced the immediate environment.

When one has recently experienced the ability to cause such a change in the environment, a person is likely to feel a high agentic ability, or, the experience of being a causal force. This could encourage an individual to make the most of any opportunities for self-driven change. However, when one experiences a low agentic ability, one may respond less to such techniques. After all, this person would be less likely to consider him- or herself to be a causal force, simultaneously increasing the susceptibility to external sources.

Previous research has linked increased long-term control beliefs to self-facilitated change [4], [5], [6]. For example, individuals with a high belief in the ability to control events in their life showed increased attitude change after writing a counter-attitudinal essay [7]. Furthermore, an individuals’ belief in the ability to control situations strongly influences the power a person has to seek out challenges [8] and effectively deal with those challenges [9]. Control beliefs are therefore of central importance to a wide range of theoretical models of behavioral change [4], [10], [11], [12], [13].

Low perceptions of control have however been linked to increased susceptibility towards others [7], [14], [15], as we may be more likely to be influenced by external forces when our sense of control is disrupted [16]. For example, individuals who in general believe having little control over the events in their lives are more likely to be influenced and persuaded by external factors than individuals with a high belief of control [14], [17]. In one of the first studies to look at the influence of state perceptions of control, Fennis and Aarts [16] showed that individuals who had recent experiences of reduced *agency* were more likely to comply with a request from the experimenter. While this study suggests that a low sense of agency seems to increase susceptibility to *direct persuasion*, the question remains whether agency can also influence the effectiveness of other persuasion techniques, such as *self-persuasion*.

Research on self-persuasion has long shown that messages that people generate to convince themselves can provide powerful and long-lasting changes [18]. Self-persuasion is more effective in changing the individual’s attitudes and behavioral intentions than arguments that are presented by others (e.g., [19]); is less prone to correction [20] or reactance [21]; and even reduces addictive behaviors like smoking [22]. The question however is under what circumstances self-persuasion is most effective; if low agency increases the effectiveness of direct persuasion techniques, can experiences of high agency then increase the effectiveness of self-persuasion techniques?

In the present study we investigated the relation between experiences of agency and the effectiveness of persuasion techniques. Participants conducted a task in which they either experienced low or high agency. Depending on experimental condition, participants then received arguments by an authority institution as to why it is important to keep the city clean, or participants were asked to generate these arguments themselves. We expected that direct persuasion would be more effective after participants had recently experienced low rather than high agency. However, we expected self-persuasion to be more effective after participants had experienced high rather than low agency.

## Method

### Participants

One-hundred-and-twenty undergraduate students (93 females; *M*_age_ = 23.33; *SD* = 2.43) from the Radboud University Nijmegen participated in exchange for course credit or €2 (Five participants took part a week later than the other participants due to lab-space considerations). They all gave written informed consent before participating in the experiment. The study was conducted in accordance with the Declaration of Helsinki and was approved by the Radboud University Nijmegen Ethics Committee of the Faculty of Social Sciences.

### Materials & procedure

Participants started with the agency manipulation task: Participants were required to press a left or a right response button on their keyboard, after which they would hear a tone presented through a headset. Participants were told that they could have produced the tone by their button-press, but that it was also possible that the computer caused the tone, thereby making the sense of agency ambiguous. In the high agency condition, participants were presented with a large number of trials in which tones were matched to specific left or right button-presses, and these tones always quickly followed the keyboard-presses. In the low agency condition, participants were presented a large number of trials in which random tones were presented, these tones took considerably longer to occur, or occurred even before presses were performed (low agency condition). Both timing and action-effect contingency have been shown to elicit strong effects on the sense of agency (e.g., [23], [24]).

Each trial started with the presentation of a row of X-es for 1500 ms in the center of the monitor. Participants were instructed to press a left or a right button the moment the fixation-cross disappeared. On high agency trials, button-presses produced a tone 100 ms after a button-press. On low agency trials, tones were produced 50 ms before the fixation-cross disappeared (therefore before a button could have been pressed), or 7750 ms after the button-press. On high agency trials left button-presses produced a 1000 Hz tone for 500 ms. A right button-press elicited a 600 Hz tone for 500 ms. On low agency trials the presented tone was a randomly selected 600 Hz or 1000 Hz tone. After tone presentation, participants had to indicate the degree to which they felt they – not the computer – had just caused the tone to occur. They could do this by clicking on a 100-point scale slider, with higher scores indicating higher feelings of causation, and lower scores indicating lower feelings of causation. The task contained 30 trials in total. The high agency condition consisted of 24 high agency and 6 low agency trials. The low agency condition consisted of 6 high agency and 24 low agency trials.

After the agency manipulation task, participants were presented with a direct persuasion or a self-persuasion manipulation, and received the following instructions:

“You are going to see a question regarding the cleanliness of the city of Nijmegen. This question will be presented on the monitor for two minutes. During that time, you will be presented with reasons which are, according the municipality of Nijmegen, the most important reasons to keep the city clean (direct persuasion condition) / during that time, write down arguments why you think it is important to keep the city clean (self-persuasion condition). Carefully attend to the question and the reasons to keep the city clean.”

In the direct persuasion condition participants were presented with 5 arguments (these arguments were mentioned most often in a pilot study; Thirty participants were asked for the most important reasons to keep the city of Nijmegen clean in a way similar to the described self-persuasion condition of the present study. The direct persuasion condition of the present study presented the responses that were most frequently given in this pilot study, which were: 1. A clean city looks and smells better; 2. A clean city is good for the environment. Waste on the street is bad for the environment; 3. A clean city is more attractive; 4. It’s important to give a good example as a society towards others, especially towards children; 5. Individuals feel happier when they walk through a clean city.), which, according to the city municipality, represented the most important reasons to keep the city of Nijmegen clean (e.g., “People feel happier when they walk through a clean city”). Arguments were presented on screen for 2 minutes. In the self-persuasion condition participants were required to type in reasons for why it is important to keep the city of Nijmegen clean. They had 2 minutes to write down their reasons. Participants generated 4.85 arguments on average (low agency condition: 4.50 vs. high agency condition 5.03; *p* = .31).

After the agency and persuasion manipulations, participants’ attitudes toward a clean environment were assessed. Participants were given 6 statements related to city and campus cleanliness (e.g., “It is important to keep the city clean”; α = .76; *M* = 7.72; *SD* = 1.27). Participants had to express agreement with each item on a scale ranging from 1 (*strongly disagree*) to 11 (*strongly agree*). Finally, participants were asked to contribute to keeping the campus clean: The experimenter allegedly worked in collaboration with the University board to stimulate environment friendly behavior. Participants were told there will be a big cleanup of the university campus in the near future in collaboration with student volunteers, and were asked whether they would be willing to participate in the campus cleanup. Participants could indicate their willingness to contribute by indicating yes or no.

Debriefings indicated that none of the participants realized the true nature of the study. Therefore, all participants were included in the analyses.

## Results

### Agency Manipulation Check

An Analysis of Variance (ANOVA) was conducted on the mean scores of the reported agency ratings, with agency condition (low agency vs. high agency) as a between-subjects predictor. Results showed that participants indeed reported increased agency in the high agency condition compared to the low agency condition (*M*_low agency_ = 17.00, *SD* = 8.10; *M*_high agency_ = 33.44, *SD* = 11.32), *F*(1, 118) = 83.66, *p* < .001, η^2^_p_ = .42. There were no differences in agency between the persuasion and self-persuasion conditions, *F* < 1, n.s.

### Attitudes ratings

**Fig 1:**
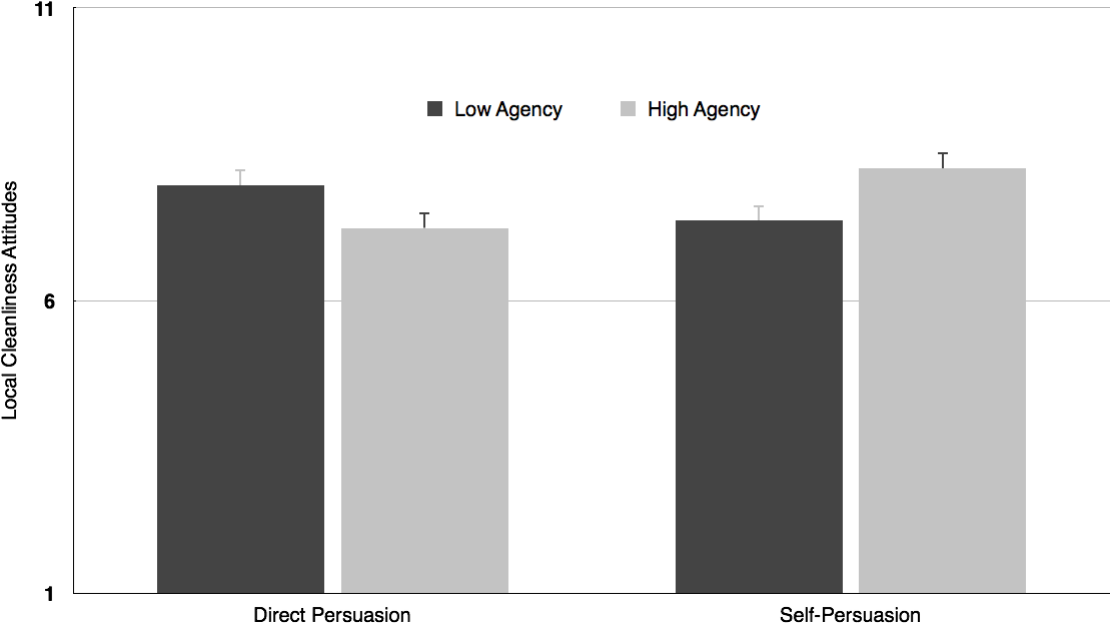
Mean cleanliness attitudes as a function of Agency and Persuasion conditions. Error bars represent standard errors.

A 2 (Agency: high vs. low) x 2 (Persuasion: direct persuasion vs. self-persuasion) ANOVA was conducted, with the mean score of the six attitude ratings towards a clean environment as dependent variable. Results showed neither a main effect of agency condition nor of persuasion condition, *F*’s < 1, n.s. However, there was a significant interaction effect between the agency and persuasion conditions, *F*(1, 116) = 9.20, *p* = .003, η^2^_p_ = .07 (See Fig 1). Simple effects showed that direct persuasion was marginally effective when participants had recently experienced low compared to high agency (*M*_low agency_ = 7.97, *SD* = 1.46; *M*_high agency_ = 7.24, *SD* = 1.41), *F*(1, 116) = 3.76, *p* = .055, η^2^_p_ = .03. However, self-persuasion was more effective after experiences of high compared to low agency (*M*_low agency_ = 7.37, *SD* = 1.63; *M*_high agency_ = 8.26, *SD* = 1.32), *F*(1, 116) = 5.51, *p* = .021, η^2^_p_ = .05. We additionally tested which persuasion technique was most effective given one’s low or high agency experiences. The simple effects showed a significant difference between the direct persuasion and self-persuasion conditions in the high agency condition, *F*(1, 116) = 7.35, *p* = .008, η^2^_p_ = .06, as self-persuasion was more effective than direct persuasion. The different persuasion techniques did not lead to statistically different results in the low agency condition, *F*(1, 116) = 2.49, *p* = .117, η^2^_p_ = .02.

**Fig 2:**
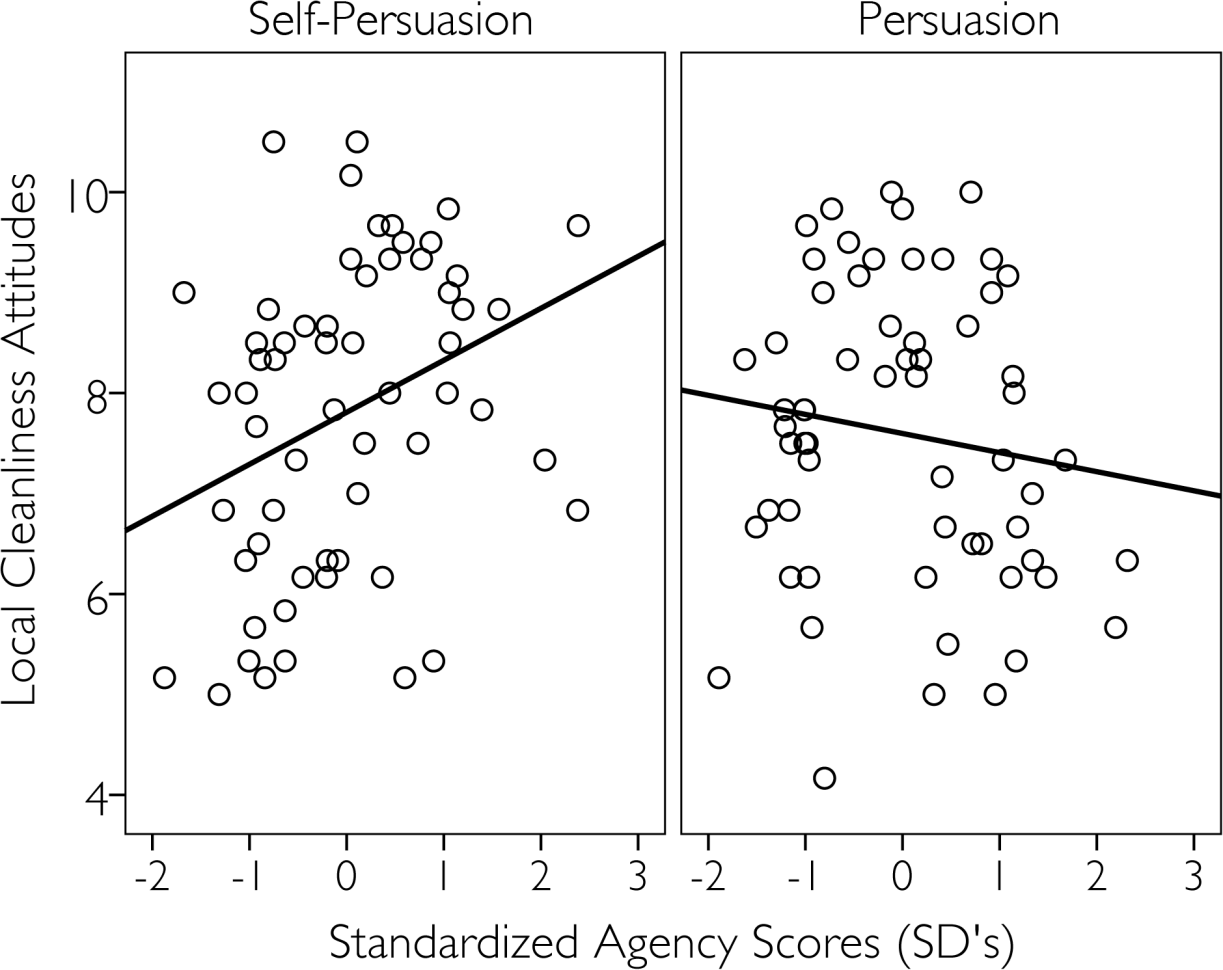
Mean cleanliness attitudes as a function of the mean standardized agency scores and the persuasion condition.

To further explore the relation between experiences of agency and effectiveness of the persuasion techniques we regressed the attitude scores on the persuasion condition and the mean agency scores (total *r* ^2^ = .07). This analysis showed neither a main effect of persuasion condition, *β =* .07, *t* (116) = .79, *p =* .432, nor did the results show a main effect of the agency scores, *β =* .11, *t* (116) = 1.21, *p =* .227. The results did however show a significant interaction between the persuasion condition and the mean agency scores, *β =* .24, *t* (116) = 2.62, *p =* .010. Simple slope analyses [25] for mean agency scores in the persuasion and self-persuasion conditions showed a significant effect in the self-persuasion condition, *β =* .35, *t* (116) = 2.62, *p =* .010. The more individuals experienced agency within the self-persuasion condition, the more they were likely to declare a clean environment was important to them (see Fig 2). The relation between agency scores and cleanliness attitudes did not reach significance within the direct persuasion condition, *β* = -.13, *t* (116) = -1.03, *p =* .303.

### Volunteering

A binary logistic regression on volunteering behavior with agency condition, persuasion condition, and the interaction between agency and persuasion as predictors was conducted (Nagelkerke *r* ^2^ = .15). Results showed only the interaction to be a significant predictor of volunteering behavior, *b* = -2.86, *SE* = .93, Wald = 9.44, *p* = .002, odds ratio = .06. Similar to the findings on cleanliness attitudes, direct persuasion led to more signups for helping out in a campus cleanup after participants had previously experienced low rather than high agency (*M*_low agency_ = .33; *M*_high agency =_ .10; *b* = -1.54, *SE* = .72, Wald = 4.57, *p* = .033, odds ratio = 0.21; means represent volunteering percentages). Within the self-persuasion condition, participants who had experienced high agency were more likely to volunteer than participants who had experienced low agency (*M*_low agency_ = .20; *M*_high agency =_ .48; *b* = 1.32, *SE* = .59, Wald = 5.01 *p* = .025, odds ratio = 3.73). In subsequent analyses we tested which persuasion technique was most effective depending one’s experiences of agency. Comparisons between the direct and self-persuasion conditions were significant in the high agency condition, *b* = -2.17, *SE* = .71, Wald = 9.24, *p* = .002, odds ratio = 0.11. Specifically, for participants with a number of high agency experiences self-persuasion was more effective than direct persuasion. The difference between the direct persuasion and self-persuasion techniques did not reach significance for participants with a number of low agency experiences, *b* = .69, *SE* = .60, Wald = 1.34, *p* = .247, odds ratio = 2.00.

We conducted another binary logistic regression using the mean agency scores (standardized) as a predictor instead of the agency condition (Nagelkerke *r* ^2^ = .09). Results showed only the interaction between the persuasion condition and the agency scores to be a significant predictor of volunteering behavior, *b* = .50, *SE* = .22, Wald = 5.08, *p* = .024, odds ratio = 1.66. There was a trend of agency scores on volunteering behavior within the self-persuasion condition, *b* = .50, *SE* = .30, Wald = 2.81, *p* = .094, odds ratio = 1.64, yet no significant effect within the direct persuasion condition, *b* = -.51, *SE* = .34, Wald = 2.33, *p* = .127, odds ratio = 0.60.

## Discussion

The present study revealed that the effectiveness of direct persuasion and self- persuasion techniques is influenced by experiences of control, specifically the sense of action control known as agency. Using a number of different analyses we investigated the influence of an agency manipulation on the degree to which different persuasion techniques were able to successfully influence attitudes and intentions. Specifically, we tested whether direct persuasion was more effective after experiences of low rather than high agency, and whether self-persuasion was more effective after experiences of high rather than low agency. Not every hypothesized difference between conditions reached a level of significance and we therefore must take care in interpreting these results, however, overall the results convincingly show differential effects of agency for the two persuasion techniques.

First, the present results showed the trend that direct persuasion is more effective after recent experiences of low agency rather than high agency, with regards to both participants’ attitudes and behavior. Similar findings were obtained by Fennis and Aarts [16] who revealed that individuals who had recently experienced weakened personal control were more likely to be susceptible to social influence attempts. The present study extends these findings by suggesting that experiences of agency not only influence behavior after a persuasive message, but can also promote attitude change. However, such an effect was not evident when we used the individual agency scores as a predictor instead of the agency manipulation, suggesting future research is needed to definitively validate the relation between agency and direct persuasion.

In prior research the effectiveness of self-persuasion has often been advocated, as it would produce more powerful and long-lasting effects than direct persuasion [18]. However, in the present study the results did not suggest a superiority of self-persuasion over direct-persuasion techniques; the degree to which self-persuasion was a powerful technique was highly dependent on the agency manipulation. This does not directly prove that self-persuasion is no longer a better technique compared to direct persuasion; perhaps individuals in general are more likely to experience at least some degree of agency, making self-persuasion generally effective tactic in daily life. The present research does however complement recent developments in research which have started to investigate the boundary conditions of self-persuasion. For example, it has been shown that requiring individuals to generate a high number of arguments (e.g., [10]) will reduce the effectiveness of the self- persuasion technique [26],[27]; and that counter-attitudinal arguments are more effective than pro-attitudinal arguments [28]. The present results add to these studies by showing that self-persuasion can be hampered or helped by experiences of agency, creating instances in which direct persuasion, at least in the short term, can be more effective than self-persuasion: When agency is low rather than high, and the sense of personal control is weakened, techniques such as self-persuasion become less powerful.

Earlier research on personality traits, such as locus of control [29] and beliefs on self-efficacy [4], demonstrated that general beliefs of personal control are related to susceptibility to persuasion [14] and to the ability for self-change [30], [7]. The present study extends these findings by showing that state-based experiences of control, such as agency, can increase susceptibility to direct persuasion after experiences of low agency rather than high agency, and increase the effectiveness of self-persuasion after experiences of high rather than low agency.

In the present study, we cannot rule out that the agency manipulation influenced alternative processes. Future studies should investigate whether experiences of agency can influence self-esteem or self-confidence, and thereby influence the effectiveness of persuasion techniques. However, given the fact that the slopes of the actual agency scores closely mapped the observed effects that we found when using the agency condition as a predictor (especially in the self-persuasion condition), suggests an important role for experiences of agency herein. A second limitation is that due to the high number of female participants, we were unable to test for possible gender differences in susceptibility to the direct or self-persuasion techniques. However, as far as we know, no such differences are reported in the relevant literature on (self)-persuasion. Finally, our design did not feature a control condition by which we were able to determine the direction of the effects. It is of course possible that only experiences of low and not high agency caused changes to occur (or vice versa). However, the ability for self-promoted change and external susceptibility have both been empirically linked to long-term beliefs about personal control (e.g., [14], [7]), and it stands to reason to assume that both processes operate in everyday life as a consequence of our experiences of agency.

While the present study focused on the sense of agency, other manipulations of control may also influence the effectiveness of (self) persuasion techniques [16]. For example, Briñol, Petty, Valle, Rucker, & Becerra [31] found that power, when induced before a message, decreased external message processing, while when power is induced after a message, a person's own thoughts exerted greater impact on their attitudes (see also [32]). The present findings extend such findings by showing the impact of recent experiences of action control, or agency.

It remains unclear exactly which process underlies the self-persuasion technique. It has been suggested that the effort in processing might be of special importance when it comes to self-persuasion [27]. Besides cognitive demand, research has also shown that correcting for externally generated stimuli is much easier [20], that individuals engaging in self-persuasion want to be seen as acting consistent with their arguments [33], or that they try to decrease cognitive dissonance [34]. Hence, more research is needed to further specify the exact mechanism.

In trying to explain his findings on destructive obedience, Stanley Milgram [15] hypothesized about an agentic shift: a lowered sense of causation that makes individuals more susceptible to powerful social heuristics such as authority figures. The present study indeed suggests that a shift in agency may influence susceptibility. Individuals were more susceptible to direct persuasion by a source of authority after experiences of low rather than high agency. A reverse effect was however found for the effectiveness of the self-persuasion technique. Here, high rather than low agency was related to an increase the effectiveness of this technique (although we would prefer it if this would not be used for the goal of destructive obedience). A number of different approaches in both the scientific and the public domain emphasize the power of self-generated change to improve individuals’ behavior, cognitions, and more general, lifestyles. The present findings suggest that with a relatively short boost of experienced control, and the prevention of situations in which personal experiences of control are weakened, we can help individuals to help themselves.
